# High Mean Corpuscular Volume Predicts Poor Outcome for Patients With Gastroesophageal Adenocarcinoma

**DOI:** 10.1245/s10434-019-07186-1

**Published:** 2019-01-31

**Authors:** Gerd Jomrich, Marlene Hollenstein, Max John, Robin Ristl, Matthias Paireder, Ivan Kristo, Reza Asari, Sebastian F. Schoppmann

**Affiliations:** 1 Department of Surgery, Medical University of Vienna, and Gastroesophageal Tumor Unit, Comprehensive Cancer Center (CCC), Vienna, Austria; 2 Section for Medical Statistics (IMS), Center of Medical Statistics, Informatics and Intelligent Systems, Medical University of Vienna, Vienna, Austria

## Abstract

**Background:**

Elevated mean corpuscular volume (MCV) is associated with a diminished prognosis for various tumor entities. This study aimed to evaluate the association between preoperative serum MCV levels and both overall (OS) and disease-free survival (DFS) for patients with resectable adenocarcinomas of the esophagogastric junction (AEG).

**Methods:**

This study included consecutive patients undergoing surgical resection between 1992 and 2016. Measured preoperative MCV levels were stratified into quintiles and correlated with patients’ survival and clinicopathologic characteristics.

**Results:**

The study analyzed 314 patients with a median OS of 36.8 months and a median DFS of 20.6 months. The multivariate analysis showed that preoperatively elevated MCV is a significant prognostic factor for OS (hazard ratio [HR], 1.05; 95% confidence interval [CI], 1.03–1.08; *P* < 0.001) and DFS (HR, 1.05; 95% CI, 1.03–1.08; *P* < 0.001). In the subgroup analysis of neoadjuvantly treated and untreated patients, MCV remained an independent prognostic factor for OS (HR, 1.08; 95% CI, 1.04–1.12; *P* < 0.001) and DFS (HR, 1.07; 95% CI, 1.03–1.12; *P* < 0.001) in both groups. In the univariate analysis, tumor stage and differentiation, adjuvant chemotherapy, MCV, mean corpuscular hemoglobin (MCH), and mean corpuscular hemoglobin concentration (MCHC) were significantly correlated with diminished OS and DFS.

**Conclusion:**

Preoperatively elevated MCV is an independent prognostic factor for patients with adenocarcinomas of the esophagus and the gastroesophageal junction.

Esophageal cancer (EC) is the sixth most common cause of cancer-related death, causing an estimated 400,000 deaths worldwide per year. In East Asian countries, EC consists mainly of esophageal squamous cell carcinoma (ESCC), located most frequently in the proximal parts of the esophagus.^1^,^2^ Whereas the number of ESCCs decreases in Western countries, the percentage of adenocarcinomas at the esophagogastric junction (AEG) is continuously increasing.^3^

Neoadjuvant (radio)chemotherapy has emerged as the current standard treatment for locally advanced AEG. Despite the development of new therapeutic approaches combining multimodal treatment protocols and surgery, the prognosis for most patients with gastroesophageal adenocarcinoma remains poor, with 5-year overall survival rates of approximately 30%0.^4^,^5^ To date, traditional tumor-based, histopathologic risk factors such as tumor and lymph node staging, tumor differentiation, and status of resection margin remain the only prognostic factors, available only retrospectively to surgery. In addition, these factors often are influenced by the use of neoadjuvant treatment.^6^,^7^ Therefore, other prognostic factors available before surgery that can evaluate prognosis and treatment response are urgently needed.

Findings have shown hematologic parameters, particularly mean corpuscular volume (MCV), to be predictive markers for patients with various cancer entities. Recently, preoperatively elevated MCV, caused by alcohol abuse and acetaldehyde and folate deficiency, was shown to be an independent prognostic factor in ESCC patients.^8^,^9^ Whereas ESCC is highly associated with alcohol and nicotine abuse, the association of alcohol consumption and the development of AEG remains unclear.^9^–^14^ Besides alcohol abuse, folate and vitamin B12 deficiency, oxidative stress, and chemotherapy are known causes for elevation and/or changes in MCV levels in cancer patients.^15^–^19^

To date, no data exist on the prognostic value of MCV for patients with gastroesophageal adenocarcinoma. In this study, we evaluated the prognostic value of preoperative MCV for patients with resectable adenocarcinomas of the esophagus and the gastroesophageal junction as well as the influence of preoperative systemic treatment on the potential prognostic value of MCV.

## Materials and Methods

### Blood Examinations

Laboratory factors including preoperative serum MCV, mean corpuscular hemoglobin (MCH), mean corpuscular hemoglobin concentration (MCHC), red cell distribution width (RDW), and hemoglobin (Hb) were obtained within 7 days before surgery. Hematologic parameters were determined using the Coulter STKS (Coulter, Hialeah, FL, USA), the Sysmex NE-8000, or the hematology analyzer Sysmex XE-2100 (both from TOA Medical Electronics, Kobe, Japan), depending on the date of blood testing, under controlled conditions at the Department of Laboratory Medicine, Medical University of Vienna, which operates as the central laboratory of the General Hospital of Vienna, a certified (ISO 9001) and accredited (ISO 15189 since 2008) quality management system.^20^–^22^

### Patients

We reviewed a prospectively maintained database of patients who underwent curative resection of locally advanced AEG between January 1992 and April 2016 in the Department of Surgery at the Medical University Vienna. Approval was obtained from the ethics committee of the Medical University Vienna, Austria, according to the declaration of Helsinki (EK1652/2016).

The exclusion criteria ruled out distant metastasis at the time of surgery, positive resection margin, postoperative death from a cause other than cancer or death within 30 days after surgery, known history of alcohol abuse, malignancies other than AEG, and missing preoperative levels of serum MCV, MCH, MCHC, RDW, and Hb. Blood for a complete blood count was drawn within 3 days before surgery. At that time, none of the patients showed signs of pyrexia (axillary ≥ 37.2 °C [99.0 °F]) or any form of active infection or chronic inflammatory disease.

The patients’ baseline clinicopathologic values were retrospectively reviewed and collected from the local database and electronic patients’ records. The pathologic classification of the primary tumor, the degree of lymph node involvement, and the presence of organ metastasis were determined according to the tumor-node-metastasis (pTNM) classification of the Union for International Cancer Control (UICC), 7th edition.

Pre- and postoperatively, every patient was discussed in the interdisciplinary tumor board meeting. Patients with resectable AEG, clinical stage T1, N1-3 or T2-4a, or N0-3 received neoadjuvant treatment. Neoadjuvant chemotherapy was performed by intravenous infusion, either with oxaliplatin/capecitabine (regimen A) or cisplatine/5-fluoruracil (regimen B) according to a current study protocol. Concomitant radiation was performed according to the recommendations of the interdisciplinary tumor board based on the regimen published by Van Hagen et al.^23^ (regimen C).

The rate of response to neoadjuvant treatment was classified as defined by Mandard et al.^24^ The tumor location at the gastroesophageal junction was classified according to Siewert and Stein.^25^

All the patients were regularly followed up with physical examination, tumor marker, and computed tomography at our outpatient clinic every 3 months for the first 2 years, then every 6 months until 5 years after surgery. Patients with missing follow-up data (lost to follow-up assessment) were excluded from analysis.

### Surgery

Transhiatal extended gastrectomy (THG) was performed for the patients with AEG 2 and 3 tumors. Proximal gastrectomy (Merendino procedure) was performed for the patients presenting with stage 1 tumors located at AEG 1 or 2 in selected cases. Abdominothoracic esophageal resection (ATE) was performed for the patients with AEG 1 or 2 tumor.

All the patients were regularly followed up with physical examination, tumor marker, and computed tomography at our outpatient clinic every 3 months for the first 2 years, then every 6 months until 5 years after surgery.

### Statistical Analysis

Statistical analysis was performed using the software R 3.3 (R Foundation for Statistical Computing, Vienna, Austria; www.r-project.org). Categorical variables were described by absolute and relative frequencies, and metric variables were described by mean and median minimum and maximum.

To analyze the association of hematologic parameters and other potential predictors with post-surgery overall survival (OS) or disease-free survival (DFS), hazard ratios corresponding to a one-unit increase in a predictor variable were estimated from uni- and multivariable Cox proportional hazard regression models. Separate models were calculated for the full sample and for the subgroups of patients with and without neoadjuvant therapy. For these analyses, UICC stages 1 and 2 as well as stages 3 and 4 were combined to ensure a sufficient sample size in each class. The reported *p* values correspond to Wald tests for the null hypothesis for a hazard ratio of 1, or in the case of UICC and AEG, for equal hazards in all factor stages. No adjustment for multiple testing was applied due to the explorative nature of the study. For visualization, preoperative MCV, MCH, MCHC, RDW, and Hb levels were stratified by sample quintiles, and Kaplan–Meier estimates of OS and DFS functions were calculated for each quintile.

## Results

In our prospective database containing 544 patients with resectable esophageal cancer, 314 patients with adenocarcinomas of the esophagogastric junction had data on preoperative hematologic parameters and were eligible for further investigation. Their mean age at time of surgery was 63.8 years (range, 30.7–89.5 years). The study included 255 male patients (81.2%). Of the 157 patients (50%) who underwent neoadjuvant treatment, 95 received regimen A, 51 received regimen B, and 11 received regimen C. The majority of the patients (*n* = 132, 43%) had clinical stage 3 disease. Of the patients receiving neoadjuvant treatment, 13 (8.3%) showed a complete response (CR).

The clinicopathologic characteristics are summarized in Table 1. The median of preoperative MCV was 91.2 fl (range, 63.5–108.2 fl). When the patients were stratified into quintiles for preoperative MCV levels, 64 patients were categorized as very low (range, 63.5–84.1 fl) and medium (range, 89.3–92.8 fl), and 62 patients were classified as low (range, 84.1–89.3 fl), high (range, 92.8–97.8 fl), and very high (range, 97.8–108 fl).

**Table 1:** Association of mean corpuscular volume (MCV) with clinicopathologic parameters in adenocarcinomas of the esophagogastric junction

Factors	All patients		Preoperative MCV		*P* value
	(*n *= 314)	%	(Mean)	SD	
Mean age: years (range)	63.8 (30.7–89.5)				NS
Sex					
Male	255	81.2	90.82	7.38	NS
Female	59	18.8	89.82	8.33	
(y) pT					
0	13	4.1	92.38	7.71	< 0.001
1	67	21.3	88.68	6.43	
2	81	25.8	88.29	7.56	
3	139	44.3	92.48	7.59	
4	4	4.5	93.51	6.95	
(y) pN					
0	131	41.7	90.58	7.19	0.018
1	113	36.0	89.60	7.92	
2	37	11.8	90.64	6.91	
3	33	10.5	94.32	7.67	
(y) G					
0	12	3.8	92.16	8.00	NS
1	6	1.9	90.12	8.32	
2	129	41.1	89.67	7.25	
3	166	52.9	91.31	7.74	
4	1	0.3	86.60		
UICC stage					
0	9	2.9	90.43	8.46	NS
1	83	26.4	88.84	6.40	
2	80	25.5	90.35	8.32	
3	132	42.0	91.94	7.52	
4	10	3.2	90.56	8.15	
AEG					
1	182	58.0	91.13	7.50	NS
2	105	33.4	89.58	7.76	
3	27	8.6	91.36	7.03	
Surgical approach					
Abdominal	115	36.6	90.28	7.04	NS
Thoracoabdominal	199	63.4	90.83	7.86	
Neoadjuvant therapy					
Yes	157	50.0	94.25	6.22	NS
No	157	50.0	87.01	7.05	
Mandard regression					
1	13	8.3	92.38	7.71	NS
2	15	9.5	93.82	3.25	
3	28	18.5	92.50	5.65	
4	50	31.8	94.07	5.74	
5	50	31.8	96.07	6.94	
Adjuvant therapy					
Yes	126	40.1	89.42	7.59	0.02
No	188	59.9	91.44	7.46	

*MCV* mean corpuscular volume, *SD* standard deviation, *NS* not significant, *UICC* Union for International Cancer Control, *AEG* adenocarcinoma of the esophagogastric junction

**Fig. 1:**
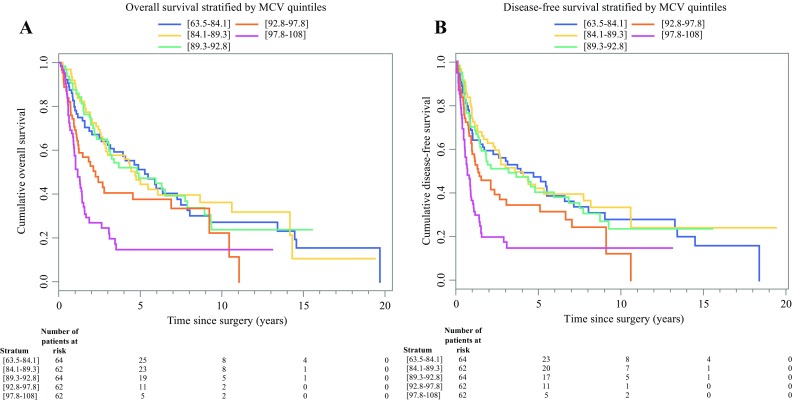
Kaplan-Meier curves for (**a**) overall survival and (**b**) disease-free survival for patients with adenocarcinoma of the esophagogastric junction (AEG) stratified by mean corpuscular volume (MCV) quintiles

For all the patients, the median OS was 36.8 months, and the median DFS was 20.6 months. The Kaplan–Meier survival analysis showed that high MCV, MCH, and MCHC all were associated with poor OS and DFS for all the patients (all *P* < 0.001) (Fig. 1a and b).

In the subgroup analysis of neoadjuvantly treated and untreated patients, elevated MCV, MCH, and MCHC remained highly associated with diminished OS and DFS as well. No significant correlation of RDW and Hb could be found for OS and DFS in any of the groups. The Cox regression analysis identified tumor stage, lymph node status, tumor differentiation, adjuvant chemotherapy, MCV, MCH, and MCHC as the significant prognostic factors for OS and DFS in all the patients.

In the subgroup analysis of neoadjuvantly treated patients, tumor stage, lymphnode status, tumor differentiation, Mandard response rate, MCV, MCH, and MCHC were significantly associated with shorter OS and DFS. In addition, sex was shown to be a significant factor for DFS in neoadjuvantly treated AEG patients.

Regression analysis using multivariate Cox models showed that the independent risk factors for OS and DFS for all the patients were tumor staging (respectively HR, 0.52; 95% CI, 0.13–2.04; *P* = 0.014 and HR, 0.63; 95% CI, 0.16–2.45; *P* = 0.004) and differentiation (respectively HR, 1.54; 95% CI, 1.16–2.03; *P* = 0.005 and HR, 1.50; 95% CI, 0.16–1.95; *P* = 0.006), adjuvant chemotherapy (respectively HR, 0.62; 95% CI, 0.45–0.86; *P* = 0.004 and HR, 0.62; 95% CI, 0.46–0.85; *P* = 0.003), MCV (respectively HR, 1.05; 95% CI, 1.03–1.08; *P* < 0.001 and HR, 1.05; 95% CI, 1.03–1.08; *P* < 0.001), MCH (respectively HR, 1.14; 95% CI, 1.07–1.22; *P* < 0.001 and HR, 1.12; 95% CI, 1.05–1.20; *P* < 0.001), and MCHC (respectively HR, 1.17; 95% CI, 1.07–1.28; *P* = 0.001 and HR, 1.17; 95% CI, 1.07–1.27; *P* < 0.001) (Table 2).

**Table 2:** Multivariate Cox regression analysis estimating the influence of hematologic and clinicopathologic parameters on overall survival (OS) and disease-free survival (DFS) for all patients

Factors	*P* value (multivariate)	HR	95% CI	
OS				
Age	0.348	1.01	0.99	1.02
Sex	0.942	1.01	0.70	1.48
UICC	0.014 (global)			
1 + 2 versus 0	0.514	2.17	0.21	22.30
3 + 4 versus 0	0.300	3.38	0.34	33.73
1–2 versus 3–4	0.007	0.64	0.47	0.89
G	0.005 (global)			
1–2 versus 0	0.540	0.53	0.07	4.10
3–4 versus 0	0.904	0.88	0.11	6.78
1–2 versus 3–4	0.001	0.60	0.44	0.81
AEG	0.565 (global)			
2 versus 1	0.585	0.91	0.64	1.29
3 versus 1	0.300	0.71	0.37	1.36
2 versus 3	0.453	1.28	0.67	2.43
Neoadjuvant therapy (no vs. yes)	0.113	1.33	0.94	1.88
Adjuvant therapy (no vs. yes)	0.004	0.62	0.45	0.86
MCV	< 0.001	1.05	1.03	1.08
MCH	< 0.001	1.14	1.07	1.22
MCHC	0.001	1.17	1.07	1.28
RDW	0.538	0.98	0.93	1.04
Hb	0.591	0.98	0.90	1.06
DFS				
Age	0.330	1.01	0.99	1.02
Sex	0.968	0.99	0.69	1.44
UICC	0.004 (global)	0.63	0.16	2.45
1 + 2 versus 0	0.791	1.37	0.13	13.97
3 + 4 versus 0	0.480	2.29	0.23	22.73
1–2 versus 3–4	< 0.001	0.60	0.44	0.82
G	0.006 (global)			
1–2 versus 0	0.993	0.99	0.13	7.59
3–4 versus 0	0.648	1.60	0.21	12.17
1–2 versus 3–4	0.002	0.62	0.46	0.83
AEG	0.501 (global)			
2 versus 1	0.484	0.89	0.64	1.24
3 versus 1	0.274	0.70	0.37	1.32
2 versus 3	0.465	1.27	0.67	2.39
Neoadjuvant therapy (no vs. yes)	0.099	1.34	0.95	1.88
Adjuvant therapy (no vs. yes)	0.003	0.62	0.46	0.85
MCV	< 0.001	1.05	1.03	1.08
MCH	< 0.001	1.12	1.05	1.20
MCHC	< 0.001	1.17	1.07	1.27
RDW	0.794	0.99	0.94	1.05
Hb	0.775	0.99	0.92	1.07

*HR* hazard ratio, *CI* confidence interval, *UICC* Union for International Cancer Control, *AEG* adenocarcinoma of the esophagogastric junction, *MCV* mean corpuscular volume, *MCH* mean corpuscular hemoglobin, *MCHC* mean corpuscular hemoglobin concentration, *RDW* red blood cell distribution width, *Hb* hemoglobin, *G* tumor differentiation

In the multivariate Cox regression analysis of the neoadjuvantly treated patients, only MCV (HR, 1.08; 95% CI, 1.04–1.12; *P* < 0.001) and MCH (HR, 1.14; 95% CI, 1.02–1.27; *P* = 0.023) remained independent prognostic factors for OS. For DFS, only MCV (HR, 1.07; 95% CI, 1.03–1.12; *P* < 0.001) was found to be an independent prognostic factor (Table 3).

**Table 3:** Multivariate Cox regression analysis estimating the influence of hematologic and clinicopathologic parameters on overall survival (OS) and disease-free survival (DFS) for neoadjuvantly treated patients

Factors	*P* value (multivariate)	HR	95% CI	
OS				
Age	0.535	0.99	0.97	1.02
Sex	0.175	0.62	0.31	1.24
UICC	0.463 (global)			
1 + 2 versus 0	0.737	1.53	0.13	18.00
3 + 4 versus 0	0.575	2.00	0.18	22.59
1 + 2 versus 3 + 4	0.270	0.76	0.47	1.23
G	0.570 (global)			
1–2 versus 0	0.721	0.65	0.06	6.68
3–4 versus 0	0.894	0.85	0.08	8.92
1–2 versus 3–4	0.309	0.77	0.46	1.28
Mandard (1–2 vs. 3–5)	0.437	1.11	0.85	1.47
AEG	0.602 (global)			
2 versus 1	0.323	1.40	0.72	2.75
3 versus 1	0.835	1.10	0.44	2.75
2 versus 3	0.599	1.27	0.52	3.14
Neoadjuvant therapy regimen	0.800 (global)			
B versus A	0.506	0.84	0.51	1.39
C versus A	0.942	0.97	0.39	2.37
B versus C	0.777	0.87	0.34	2.23
Adjuvant therapy (no vs. yes)	0.813	1.06	0.65	1.72
MCV	< 0.001	1.08	1.04	1.12
MCH	0.023	1.14	1.02	1.27
MCHC	0.343	1.10	0.90	1.35
RDW	0.833	0.99	0.91	1.08
Hb	0.670	0.97	0.85	1.11
DFS				
Age	0.451	0.99	0.97	1.01
Sex	0.128	0.59	0.30	1.17
UICC	0.444 (global)			
1 + 2 versus 0	0.985	0.98	0.09	11.07
3 + 4 versus 0	0.826	1.31	0.12	14.27
1 + 2 versus 3 + 4	0.209	0.75	0.47	1.18
G	0.871 (global)			
1–2 versus 0	0.961	0.94	0.10	9.11
3–4 versus 0	0.954	1.07	0.11	10.44
1–2 versus 3–4	0.599	0.88	0.56	1.40
Mandard (1–2 vs. 3–5)	0.104	1.24	0.96	1.61
AEG	0.634 (global)			
2 versus 1	0.363	1.34	0.71	2.53
3 versus 1	0.912	1.05	0.42	2.63
2 versus 3	0.586	1.27	0.53	3.06
Neoadjuvant therapy regimen	0.609 (global)			
B versus A	0.653	0.90	0.56	1.43
C versus A	0.338	0.65	0.27	1.56
B versus C	0.491	1.37	0.56	3.40
Adjuvant therapy (no vs. yes)	0.481	0.85	0.54	1.34
MCV	< 0.001	1.07	1.03	1.12
MCH	0.059	1.10	1.00	1.23
MCHC	0.174	1.16	0.94	1.42
RDW	0.788	1.01	0.93	1.09
Hb	0.729	0.98	0.86	1.11

*HR* hazard ratio, *CI* confidence interval, *UICC* Union for International Cancer Control, *AEG* adenocarcinoma of the esophagogastric junction, *MCV* mean corpuscular volume, *MCH* mean corpuscular hemoglobin, *MCHC* mean corpuscular hemoglobin concentration, *RDW* red blood cell distribution width, *Hb* hemoglobin, *G* tumor differentiation

In the multivariate subgroup analysis of non-neoadjuvantly treated patients, the independent prognostic factors for OS and DFS were found to be tumor differentiation (respectively HR, 1.55; 95% CI, 1.01–2.40; *P* = 0.04 and HR, 1.69; 95% CI, 1.09–2.61; *P* = 0.018), adjuvant chemotherapy (respectively HR, 0.33; 95% CI, 0.18–0.60; *P* < 0.001 and HR, 0.37; 95% CI, 0.20–0.67; *P* < 0.001), MCV (respectively HR, 1.04; 95% CI, 1.01–1.08; *P* = 0.01 and HR, 1.05; 95% CI, 1.01–1.08; *P* = 0.005), MCH (respectively HR, 1.13; 95% CI, 1.03–1.24; *P* = 0.011 and HR, 1.11; 95% CI, 1.01–1.21; *P* = 0.03), and MCHC (respectively HR, 1.19; 95% CI, 1.06–1.33; *P* = 0.002 and HR, 1.18; 95% CI, 1.06–1.31; *P* = 0.003) (Table 4).

**Table 4:** Multivariate Cox regression analysis estimating the influence of hematologic and clinicopathologic parameters on overall survival (OS) and disease-free survival (DFS) for neoadjuvantly untreated patients

Factors	*p* value (multivariate)	HR	95% CI	
OS				
Age	0.115	1.02	1.00	1.04
Sex	0.216	1.35	0.84	2.16
UICC (3–4 vs. 1–2)	0.494	1.20	0.71	2.00
G (3–4 vs. 1–2)	0.047	1.55	1.01	2.40
AEG	0.862 (global)			
2 versus 1	0.689	0.92	0.60	1.40
3 versus 1	0.649	0.77	0.24	2.42
2 versus 3	0.753	1.20	0.39	3.71
Adjuvant therapy (no vs. yes)	< 0.001	0.33	0.18	0.60
MCV	0.010	1.04	1.01	1.08
MCH	0.011	1.13	1.03	1.24
MCHC	0.002	1.19	1.06	1.33
RDW	0.413	0.97	0.91	1.04
Hb	0.998	1.00	0.89	1.12
DFS				
Age	0.047	1.02	1.00	1.04
Sex	0.082	1.53	0.96	2.46
UICC (3–4 vs. 1–2)	0.268	1.34	0.80	2.26
G (3–4 vs. 1–2)	0.018	1.69	1.09	2.61
AEG	0.936 (global)			
2 versus 1	0.725	0.93	0.62	1.40
3 versus 1	0.992	1.01	0.32	3.14
2 versus 3	0.890	0.92	0.30	2.83
Adjuvant therapy (no vs. yes)	< 0.001	0.37	0.20	0.67
MCV	0.005	1.05	1.01	1.08
MCH	0.025	1.11	1.01	1.21
MCHC	0.003	1.18	1.06	1.31
RDW	0.464	0.97	0.91	1.04
Hb	0.853	1.01	0.91	1.13

*HR* hazard ratio, *CI* confidence interval, *UICC* Union for International Cancer Control, *AEG* adenocarcinoma of the esophagogastric junction, *MCV* mean corpuscular volume, *MCH* mean corpuscular hemoglobin, *MCHC* mean corpuscular hemoglobin concentration, *RDW* red blood cell distribution width, *Hb* hemoglobin, *G* tumor differentiation

## Discussion

High MCV plasma levels were significantly correlated with diminished OS and DFS, defining the preoperative MCV plasma level as an independent prognostic factor for patients with adenocarcinomas of the esophagus and the gastroesophageal junction. Besides MCV, the prognostic role of the MCH, MCHC, RDW, and Hb levels was evaluated, but only MCH, MCHC, and RDW could be shown as independent prognostic factors for OS and DFS.

Anemia is common among cancer patients, and the prognostic role of hematologic parameters has been reported for a number of malignancies, including ESCC.^9^ However, to date, no data exist on the correlation between MCV and the survival of patients with adenocarcinomas of the esophagus and the gastroesophageal junction. Because MCV is an indicator for the red blood cell volume, it often is used for detection of megaloblastic- or iron-deficiency anemia. Macrocytosis often is related to liver and blood disease, alcohol consumption, smoking, and vitamin B12 or folate deficiency.^26^–^28^

In contrast to ESCC, no correlation exists between alcohol consumption and the development of AEG. Therefore, other reasons for elevated MCV must exist in patients with adenocarcinomas of the esophagus and the gastroesophageal junction. A potential explanation might be the location of the tumor at the gastroesophageal junction. Malignant tumors located near the cardia of the stomach, developing from chronic atrophic gastritis, could result in an impaired function of the parietal cells and therefore could lead to vitamin B12 deficiency and macrocytic anemia.

Another cause for vitamin B12 deficiency and macrocytic anemia might be malnutrition, a negative prognostic factor in various malignancies, including esophageal cancer.^29^–^31^ Due to dysphagia, a common symptom of patients with adenocarcinomas of the esophagus and the gastroesophageal junction, impaired nutritional intake leads to lowered serum concentration of electrolytes, glucose, and proteins, causing decreased crystal osmotic pressure, which leads to red cell dilation (macrocytosis).^32^

Recently, we showed that malnutrition and inflammation are independent prognostic factors in AEG.^31^ Therefore, malnutrition and inflammation (systemic inflammatory response [SIR]) might provide another potential explanation for the association among increased MCV and cancer mortality.

Besides factors such as blood loss, hemolysis, tumor infiltration, malnutrition, and chronic cytokine-related anemia, chemotherapy and radiation are known reasons for anemia in cancer patients.^17^ Neoadjuvant chemo(radio)therapy in combination with surgical resection has become the current standard regimen for locally advanced AEG.^33^,^34^

We therefore investigated the prognostic role of hematologic parameters in a subgroup of neoadjuvantly treated patients with adenocarcinomas of the esophagus and the gastroesophageal junction. In the multivariate analysis, we found that MCV and MCH were independent prognostic factors for OS and that MCV was an independent prognostic factor for DFS in neoadjuvantly treated patients with adenocarcinomas of the esophagus and the gastroesophageal junction.

Interestingly, a number of studies have reported a prognostic benefit for patients experiencing macrocytosis during chemotherapy.^17^,^19^ In contrast, our findings and previously published data by other study groups show that preoperative elevated serum MCV is associated with diminished patient survival.^9^,^15^ A possible explanation might be that for patients experiencing macrocytosis during chemotherapy, not the elevated MCV, but the longer observation period until development of macrocytosis is the factor resulting in a survival benefit. On the other hand, it is remarkable that MCV was an independent prognostic factor in both of our investigated subgroups (neoadjuvantly treated and untreated patients), whereas no statistical significance could be found for UICC stage and neoadjuvant treatment.

Although our finding that UICC stage is not significantly associated with patient outcome accords well with recently published data by Anderegg et al.^35^ it remains unclear why neoadjuvant treatment was not a prognostic factor for patient survival in this study. Although response rates after neoadjuvant treatment vary throughout published data (5–29%), the response rate of only 8.3% in our study was not as high as the rates reported in previously published studies.^23^,^36^

Our finding that MCV, but not RDW or Hb, is a prognostic factor independent of other (hematologic) parameters for patients with adenocarcinomas of the esophagus and the gastroesophageal junction accords well with the data published by Yoon et al.^15^ underscoring the prognostic potential of a number of possible hematologic prognostic markers.

Although we could show that MCV is an independent prognostic factor in AEG, this study had certain limitations. Besides its retrospective nature, the long observation period must be addressed. During this period, both the surgical techniques and the regimens of neoadjuvant treatment undergo significant changes. Those two points cannot be disregarded, although the surgical approach and neoadjuvant therapy were factors that did not show any significant correlation in our study.

Based on our findings, clinicians might well focus on identifying patients at high risk for early recurrence and diminished survival after surgery. The possible consequences of preoperatively elevated MCV might be that patients receive neoadjuvant treatment despite their low clinical staging and must followed up in shorter intervals after surgery or receive adjuvant chemotherapy even though they show no lymph node involvement in their pathologic staging.

Nevertheless, the mechanisms for the development of macrocytosis and therefore the background for the prognostic role of MCV are not fully understood to date. Thus, further studies investigating preoperative vitamin B12 and folate status are needed to clarify the prognostic role of MCV for patients with adenocarcinomas of the esophagus and the gastroesophageal junction.

## References

[1] Ferlay J, Soerjomataram I, Dikshit R (2015). Cancer incidence and mortality worldwide: sources, methods, and major patterns in GLOBOCAN 2012. Int J Cancer..

[2] Smyth EC, Lagergren J, Fitzgerald RC (2017). Oesophageal cancer. Nat Rev Dis Primers..

[3] Arnold M, Soerjomataram I, Ferlay J, Forman D (2015). Global incidence of oesophageal cancer by histological subtype in 2012. Gut..

[4] Fontana E, Smyth EC, Cunningham D (2016). Esophagogastric adenocarcinoma: is more chemotherapy better?. Curr Treat Options Oncol..

[5] Shapiro J, van Lanschot JJB, Hulshof M (2015). Neoadjuvant chemoradiotherapy plus surgery versus surgery alone for oesophageal or junctional cancer (CROSS): long-term results of a randomised controlled trial. Lancet Oncol..

[6] Brown CS, Gwilliam N, Kyrillos A (2018). Predictors of pathologic upstaging in early esophageal adenocarcinoma: results from the national cancer database. Am J Surg..

[7] Ayez N, Lalmahomed ZS, van der Pool AE (2011). Is the clinical risk score for patients with colorectal liver metastases still useable in the era of effective neoadjuvant chemotherapy?. Ann Surg Oncol..

[8] Nagai H, Yuasa N, Takeuchi E, Miyake H, Yoshioka Y, Miyata K (2018). The mean corpuscular volume as a prognostic factor for colorectal cancer. Surg Today..

[9] Zheng YZ, Dai SQ, Li W (2013). Prognostic value of preoperative mean corpuscular volume in esophageal squamous cell carcinoma. World J Gastroenterol..

[10] Freedman ND, Abnet CC, Leitzmann MF (2007). A prospective study of tobacco, alcohol, and the risk of esophageal and gastric cancer subtypes. Am J Epidemiol..

[11] Hazelton WD, Curtius K, Inadomi JM (2015). The role of gastroesophageal reflux and other factors during progression to esophageal adenocarcinoma. Cancer Epidemiol Biomarkers Prev..

[12] Wang JH, Luo JY, Dong L, Gong J, Tong M (2004). Epidemiology of gastroesophageal reflux disease: a general population-based study in Xi’an of Northwest China. World J Gastroenterol..

[13] Chari S, Teyssen S, Singer MV (1993). Alcohol and gastric acid secretion in humans. Gut..

[14] Lubin JH, Cook MB, Pandeya N (2012). The importance of exposure rate on odds ratios by cigarette smoking and alcohol consumption for esophageal adenocarcinoma and squamous cell carcinoma in the Barrett’s Esophagus and Esophageal Adenocarcinoma Consortium. Cancer Epidemiol..

[15] Yoon HJ, Kim K, Nam YS, Yun JM, Park M (2016). Mean corpuscular volume levels and all-cause and liver cancer mortality. Clin Chem Lab Med..

[16] Kaferle JSC (2009). Evaluation of macrocytosis. Am Fam Physician..

[17] Jung HA, Kim HJ, Maeng CH (2015). Changes in the mean corpuscular volume after capecitabine treatment are associated with clinical response and survival in patients with advanced gastric cancer. Cancer Res Treat..

[18] Karvellas CJ, Sawyer M, Hamilton M, Mackey JR (2004). Effect of capecitabine on mean corpuscular volume in patients with metastatic breast cancer. Am J Clin Oncol..

[19] Wenzel C, Mader RM, Steger GG (2003). Capecitabine treatment results in increased mean corpuscular volume of red blood cells in patients with advanced solid malignancies. Anticancer Drugs..

[20] Haslacher H, Szekeres T, Gerner M (2017). The effect of storage temperature fluctuations on the stability of biochemical analytes in blood serum. Clin Chem Lab Med..

[21] Ruzicka K, Veitl M, Thalhammer-Scherrer R, Schwarzinger I (2001). The new hematology analyzer Sysmex XE-2100: performance evaluation of a novel white blood cell differential technology. Arch Pathol Lab Med..

[22] Brigden ML, Page NE, Graydon C (1993). Evaluation of the Sysmex NE-8000 automated hematology analyzer in a high-volume outpatient laboratory. Am J Clin Pathol..

[23] van Hagen P, Hulshof MC, van Lanschot JJ (2012). Preoperative chemoradiotherapy for esophageal or junctional cancer. N Engl J Med..

[24] Mandard AM, Dalibard F, Mandard JC (1994). Pathologic assessment of tumor regression after preoperative chemoradiotherapy of esophageal carcinoma: clinicopathologic correlations. Cancer..

[25] Siewert JR, Stein HJ (1998). Classification of adenocarcinoma of the oesophagogastric junction. Br J Surg..

[26] Kaferle J, Strzoda CE (2009). Evaluation of macrocytosis. Am Fam Physician..

[27] Oh RC, Holt SN, Hitchcock K, Hoekzema G (2008). How do you evaluate macrocytosis without anemia?. J Fam Pract..

[28] Yokoyama A, Yokoyama T, Kumagai Y (2005). Mean corpuscular volume, alcohol flushing, and the predicted risk of squamous cell carcinoma of the esophagus in cancer-free Japanese men. Alcohol Clin Exp Res..

[29] Espinosa E, Feliu J, Zamora P (1995). Serum albumin and other prognostic factors related to response and survival in patients with advanced non-small cell lung cancer. Lung Cancer..

[30] Onate-Ocana LF, Aiello-Crocifoglio V, Gallardo-Rincon D (2007). Serum albumin as a significant prognostic factor for patients with gastric carcinoma. Ann Surg Oncol..

[31] Jomrich G, Hollenstein M, John M (2018). The modified Glasgow prognostic score is an independent prognostic indicator in neoadjuvantly treated adenocarcinoma of the esophagogastric junction. Oncotarget..

[32] Porath-Furedi A (1983). The mutual effect of hydrogen ion concentration and osmotic pressure on the shape of the human erythrocyte as determined by light scattering and by electronic cell volume measurement. Cytometry..

[33] Burmeister BH, Smithers BM, Gebski V (2005). Surgery alone versus chemoradiotherapy followed by surgery for resectable cancer of the oesophagus: a randomised controlled phase III trial. Lancet Oncol..

[34] Reynolds JV, Muldoon C, Hollywood D (2007). Long-term outcomes following neoadjuvant chemoradiotherapy for esophageal cancer. Ann Surg..

[35] Anderegg MCJ, van der Sluis PC, Ruurda JP (2017). Preoperative chemoradiotherapy versus perioperative chemotherapy for patients with resectable esophageal or gastroesophageal junction adenocarcinoma. Ann Surg Oncol..

[36] Wright CD, Mathisen DJ, Wain JC (1994). Evolution of treatment strategies for adenocarcinoma of the esophagus and gastroesophageal junction. Ann Thorac Surg..

